# A Novel Biomolecule-Mediated Reduction of Graphene Oxide: A Multifunctional Anti-Cancer Agent

**DOI:** 10.3390/molecules21030375

**Published:** 2016-03-18

**Authors:** Yun-Jung Choi, Eunsu Kim, Jae Woong Han, Jin-Hoi Kim, Sangiliyandi Gurunathan

**Affiliations:** Department of Stem Cell and Regenerative Biology, Konkuk University, Seoul 143-701, Korea; choi_yunjung@nate.com (Y.-J.C.); np-gennao@hanmail.net (E.K.); woong1211@naver.com (J.W.H.); jhkim541@konkuk.ac.kr (J.-H.K.)

**Keywords:** uric acid, graphene oxide, reduced graphene oxide, cell viability, ovarian cancer cells

## Abstract

Graphene oxide (GO) is a monolayer of carbon atoms that form a dense honeycomb structure, consisting of hydroxyl and epoxide functional groups on the two accessible sides and carboxylic groups at the edges. In contrast, graphene is a two-dimensional sheet of sp^2^-hybridized carbon atoms packed into a honeycomb lattice. Graphene has great potential for use in biomedical applications due to its excellent physical and chemical properties. In this study, we report a facile and environmentally friendly approach for the synthesis of reduced graphene oxide (rGO) using uric acid (UA). The synthesized uric acid-reduced graphene oxide (UA-rGO) was fully characterized by ultraviolet-visible (UV-Vis) absorption spectra, X-ray diffraction (XRD), dynamic light scattering (DLS), Fourier transform infrared (FTIR), scanning electron microscopy (SEM), and Raman spectroscopy. GO and UA-rGO induced a dose-dependent decrease in cell viability and induced cytotoxicity in human ovarian cancer cells. The results from this study suggest that UA-rGO could cause apoptosis in mammalian cells. The toxicity of UA-rGO is significantly higher than GO. Based on our findings, UA-rGO shows cytotoxic effects against human ovarian cancer cells, and its synthesis is environmentally friendly. UA-rGO significantly inhibits cell viability by increasing lactate dehydrogenase (LDH) release, reactive oxygen species (ROS) generation, activation of caspase-3, and DNA fragmentation. This is the first report to describe the comprehensive effects of UA-rGO in ovarian cancer cells. We believe that the functional aspects of newly synthesized UA-rGO will provide advances towards various biomedical applications in the near future.

## 1. Introduction

Graphene, a novel two-dimensional nanomaterial composed of sp^2^-bonded carbon atoms, possesses unique physical and chemical properties including electronic, conductivity, optical, thermal, and mechanical properties [1,2,3,4,5,6]. Due to the versatile applications of graphene and its derivatives, a high demand exists for mass production of graphene through reduction of GO using various methods [7,8]. Chemical reduction appears very simple, but it usually generates graphene-like film exhibiting a relatively low C:O ratio and a considerable amount of residual functional groups, resulting in a highly resistive film [9,10].

A number of studies have reported GO reduction using various bio-molecules such as ascorbic acid [11], amino acids [12], glucose [4,13], bovine serum albumin [14], melatonin [15], humanin [16], enhanced green fluorescent protein [17] and resveratrol [18]. In the search for a novel material for the bio-reduction of GO, we found that uric acid (UA), an anti-oxidant molecule, had not been explored. UA is a byproduct of the metabolic breakdown of purines from human waste and it contains carbon, nitrogen, oxygen, and hydrogen. Therefore, in this study, we selected UA for GO reduction.

Carbon nanomaterials, graphene, and its derivatives have several biomedical applications; thus, a systematic evaluation of their potential toxicity to mammalian cells is critically important [19,20]. Several studies on the toxicity of graphene and GO in bacterial species have been reported. Studies on bacterial toxicity are essential to develop graphene as an antimicrobial agent or coating or products. Akhavan *et al.* [21] studied the bacterial toxicity of GO and rGO nanowalls against the bacteria *E. coli* and *Staphylococcus aureus*. Both graphene derivatives were effective as antibacterial materials through direct contact between the extremely sharp edges of graphene sheets and the cell wall membrane of the bacteria. Hu *et al.* [22] showed the effect of graphene on time and dose-dependent metabolic activity of *E. coli*. Liu *et al.* [23] also demonstrated the antibacterial activity of Gt, graphite oxide, GO, and rGO via membrane and oxidative stress in *E. coli*. Similarly, Gurunathan and coworkers [24,25] demonstrated the potential toxicity of GO and rGO in *E. coli* and *Pseudomonas aeruginosa* through induced production of oxidative stress in the presence of graphene materials.

The toxicity of graphene or GO sheets has been evaluated in different cell lines, including lung epithelial cells, fibroblasts, neuronal cells, and cancer cells. Chang *et al.* [26] showed that a low concentration of GO induces neither cytotoxicity nor significant cellular uptake of GO in A549 adenocarcinoma human epithelial cells. However, at higher concentrations, GO induces oxidative stress. Zhang *et al.* [27] reported the toxicity of different types of carbon nanomaterials, including nanodiamonds, carbon nanotubes, and GO in HeLa cells. They found that the lowest cellular uptake of GO, nanodiamonds, and carbon nanotubes exhibited a dose-dependent toxicity. We found that biologically reduced GO induces greater toxicity in human breast cancer cells [28,29] and ovarian cancer cells [18]. Size-dependent cytotoxic and genotoxic effects of reduced graphene oxide nanoplatelets (rGONPs) were observed in human mesenchymal stem cells [30]. Wang *et al.* [31] reported that GO would induce remarkable cytotoxicity of human fibroblast cells at a concentration above 50 mg/L. In addition to the *in vitro* effect of GO and rGO, several researchers demonstrated the *in vivo* tumor uptake and photothermal therapy with PEGylated GO using xenograft tumor mouse models. They found a very high tumor uptake of the PEG-modified GO due to highly efficient tumor passive targeting of GO caused by EPR effect [32]. Zhang *et al.* [33] showed the antitumor effect of NGO-PEG-DOX by combination of photothermal- and chemotherapies. The combined chemo-photothermal therapy exhibited a synergistic effect that led to better cancer-killing effect than chemotherapy or photothermal therapy alone. Akhavan *et al.* [13] demonstrated that when GO was reduced and functionalized by glucose in the presence of Fe catalyst, it was biocompatible with an excellent near infrared (NIR) photothermal therapy efficiency, compared to hydrazine-reduced GO, single-wall and multi-wall carbon nanotube suspensions.

Ovarian cancer is the most lethal gynecologic malignancy [34]. Although early detection and new therapeutic approaches have been developed, the mortality rate is still increasing because the origin and pathogenesis of epithelial ovarian cancer are poorly understood [34]. Ovarian conservation appears to be particularly important for a woman’s health [34]. Although many cancer drugs dramatically reduce the size of tumors, most cancers eventually relapse, which is a very important problem to overcome [35]. Mostly women affected by this ovarian cancer over the age of 50, and it accounts for approximately 3%. Most ovarian cancer cells are initially chemosensitive and later it develops chemoresistance [35]. Hence, it is necessary to identify other possible therapeutic approaches to reduce the mortality rate of this devastating disease. Therefore, the challenge is to identify cost-effective, sensitive lead molecules that have target cell specificity and increase the sensitivity. To address the anticancer activity of UA-rGO, UA is a major antioxidant in human plasma; abnormal concentrations of UA have been linked to several diseases including obesity, hypertension, cardiovascular disease, and conditions associated with oxidative stress [36]. UA has proposed roles in the central nervous system, particularly in conditions such as multiple sclerosis, Parkinson’s disease, and acute stroke [37].

Therefore, the objective of this study was to develop a simple, dependable, and time-saving approach for green reduction and functionalization of GO using UA. Furthermore, we conducted a comprehensive evaluation of GO and UA-reduced GO (UA-rGO) toxicity by analyzing cell viability, membrane integrity, reactive oxygen species (ROS) generation, and apoptosis in the ovarian cancer cell line A2780. The results strongly suggest that UA-mediated reduction of graphene oxide provides excellent toxicity and highly effective apoptotic activity against ovarian cancer cell line. The novel bio-molecule mediated reduced graphene oxide could open a new avenue for using natural human waste products such as UA as non-toxic substitutes for various chemical agents used for production of graphene and the resulting product could be used for anticancer therapy.

## 2. Results and Discussion

### 2.1. Synthesis and Characterization of UA-rGO by UV-Vis Spectroscopy

**Figure 1:**
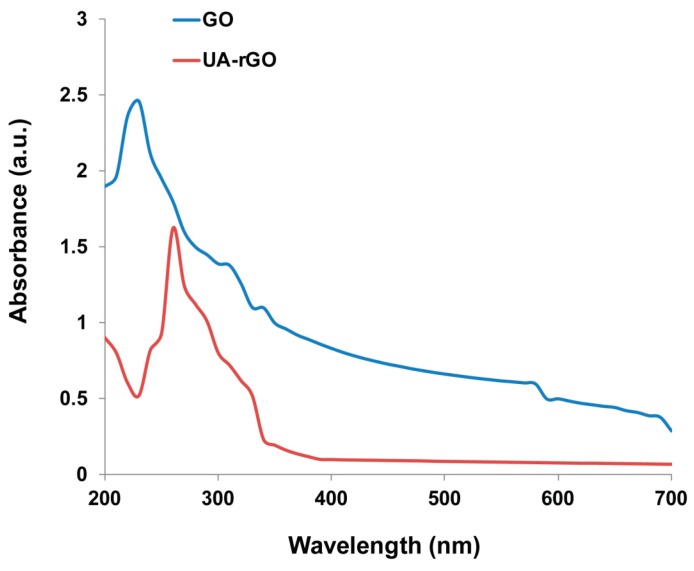
Synthesis and characterization of GO and UA-rGO by ultraviolet-visible spectroscopy. Spectra of GO exhibited a maximum absorption peak at approximately 230 nm, which corresponds to a π–π transition of aromatic C–C bonds. The absorption peak for reduced GO was red-shifted to 260 nm. At least three independent experiments were performed for each sample, and the results were reproducible. This figure shows the results of a representative experiment.

Synthesis of GO was performed by mixing Gt with potassium permanganate and sulfuric acid using a modified Hummers method, which produces readily soluble and stable colloidal suspensions of thin sheets in water [38,39,40]. Addition of UA as a reducing agent and stabilizing agent enables the GO sheets that are homogenously dispersed in water to turn from brownish to black, which provides further evidence for the reduction of GO to graphene [9]. After reduction of GO by UA, the color of the GO suspension changed from light brown to black. The black color of the rGO indicates removal of oxygen-containing bonds, resulting in electronic conjugation within reduced sheets [28,41]. Figure 1 shows the ultraviolet-visible spectra of GO and UA-rGO. The spectrum of GO has an absorption peak at 230 nm, which is shifted to 260 nm for UA-rGO. A shoulder at 290–300 nm reflects the π–π^∗^ (aromatic) type electron transitions in carbonyl groups. Two kinds of C=O groups can contribute to n π^∗^ (carbonyl) absorbance: the C=O component of the terminal carboxyl groups and the ketone groups originating from ionization and transformation of tertiary phenol OH groups on the basal plane of GO sheets [42,43]. This is a red shift, which is due to the electronic configuration of graphene in the reduction of GO. The absorption peak of GO at 230 nm is attributed to π–π^∗^ transition of aromatic C–C ring, while the absorption peak of UA-rGO at 260 nm is attributed to *n*–π^∗^ transition of C–O bonds. The phenomenon of red shift has been used as an indicator of GO reduction [18]. A similar trend was observed with various chemical and biological reagents involved in reduction of GO such as glucose [4,13], humanin [16], trimethylamine [44], and resveratrol [18].

### 2.2. X-ray Diffraction (XRD) Analysis of GO and UA-rGO

**Figure 2:**
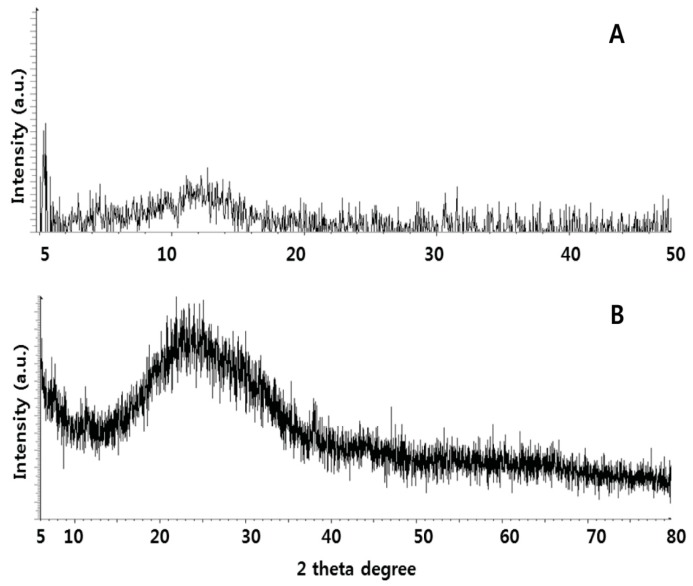
XRD patterns of GO and UA-rGO. In the XRD pattern of GO (**A**); a strong sharp peak at 2θ = 10.8° corresponds to an interlayer distance of 7.6 Å. UA-rGO (**B**) has a broad peak centered at 2θ = 25.9°, which corresponds to an interlayer distance of 3.6 Å. The changes in XRD are related to GO reduction by UA and to the removal of intercalated water molecules and oxide groups. At least three independent experiments were performed for each sample and reproducible results were obtained. Representative results are shown here.

XRD of GO and UA-rGO, synthesized by bio-molecule-mediated reduction of GO, is shown in Figure 2. The spectra in Figure 2A,B show two peaks corresponding to GO and UA-rGO. The strong peak at 2θ = 10.8° corresponds to an interlayer spacing of about 0.76 nm, indicating the presence of an oxygen functional group, which facilitated the hydration and exfoliation of graphene sheets in aqueous media (Figure 2A). After UA reduction, the hydrophilicity of water-dispersed GO sheets gradually decreased, leading to an irreversible agglomeration of rGO sheets. This peak completely disappeared after UA reduction, and a new peak at 2θ = 25.9° emerged due to graphene diffraction [45]. Thus, it can be concluded that UA effectively reduced GO to graphene. The broad peak of UA-rGO at 2θ = 25.9° indicates the exfoliation and reduction processes of GO and the processes of removing intercalated water molecules and the oxide groups (Figure 2B). This peak corresponds to 002 plane of Gt with interlayer spacing of 0.35 nm, which is due to the removal of oxygen atoms that got into the Gt gallery during the intercalation process [40]. The results also suggest that the interlayer spacing is very close to pristine graphite, indicating that the functional groups of GO have been efficiently removed [24]. The decreased interlayer suggests that removal of oxygen and water from the interlayer occurred during exfoliation, at a large rate [24]. Though there is a decrease in the interlayer spacing compared with GO, the basal spacing of UA-rGO is higher than that of well-ordered graphite (single-layer pristine graphene). The higher basal spacing may be due to the presence of residual oxygen functional groups, indicating incomplete reduction of GO [24]. However, a new broad diffraction halo was observed near 2θ = 25.9° instead of the highly symmetrical diffraction peak at 2θ = 25.9° after strong reduction, which indicates crystallisation degree of graphite declined dramatically.

The results suggest that the properties of UA-converted graphene nanosheets are comparable to those of graphene nanosheets that were chemically reduced using biological molecules such as ascorbic acid [46], amino acids [47], and plant extracts [48,49,50]. Our data indicate that GO reduction can be performed using UA, which is in agreement with earlier studies using biological systems such as wild carrot root [51], Baker’s yeast [52], *E. coli* [53], and *Bacillus marisflavi* [28].

### 2.3. FTIR Spectra of GO and UA-rGO

**Figure 3:**
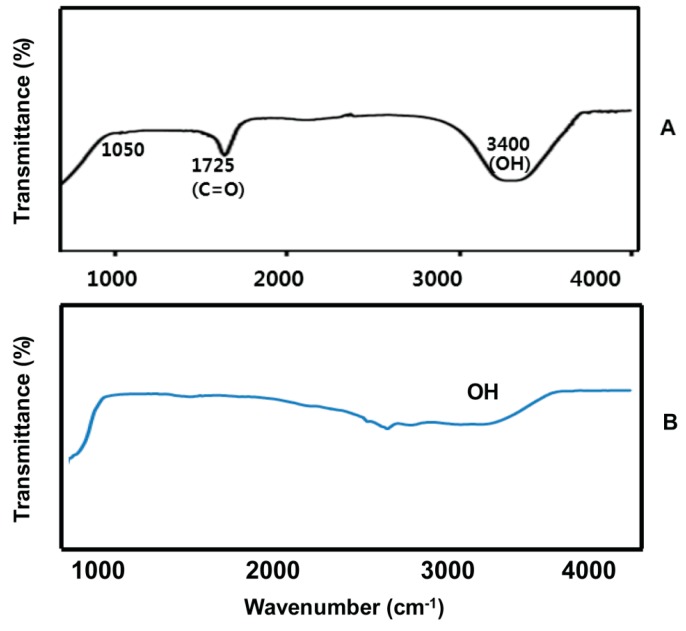
FTIR spectra of GO (**A**) and UA-rGO (**B**). FTIR spectrum of the UA-rGO shows significant reduction of nearly all of the oxygen-containing functional groups, especially the carboxylic acid groups and epoxide bonds.

The structural analysis of the GO and UA-rGO was performed using FTIR spectroscopy in the wave number ranging from 500 to 4000 cm^−1^, as shown in Figure 3A,B. The most prominent peaks were observed in the spectrum of GO at 1725 cm^−1^, due to the presence of C=O carbonyl stretching, and at 3400 cm^−1^, due to the presence of OH. These important oxygen functional groups are a characteristic feature of GO (Figure 3A). Interestingly, after the UA reduction the stretching vibration of C=O had disappeared, and other oxygen-containing functional groups in GO also decreased dramatically (Figure 3B). The spectrum shows a peak of UA-rGO representing O–H stretching vibrations observed at 3400 cm^−1^, which were significantly reduced due to deoxygenation [54,55]. The results clearly suggest that UA successfully reduced GO to graphene. Hence, the FTIR spectra indicate that the bulk of the oxygen-containing functional groups were reduced significantly from GO, and it further confirming the reduction of GO by biological means.

### 2.4. Size Analysis of GO and UA-rGO by Dynamic Light Scattering (DLS)

**Figure 4:**
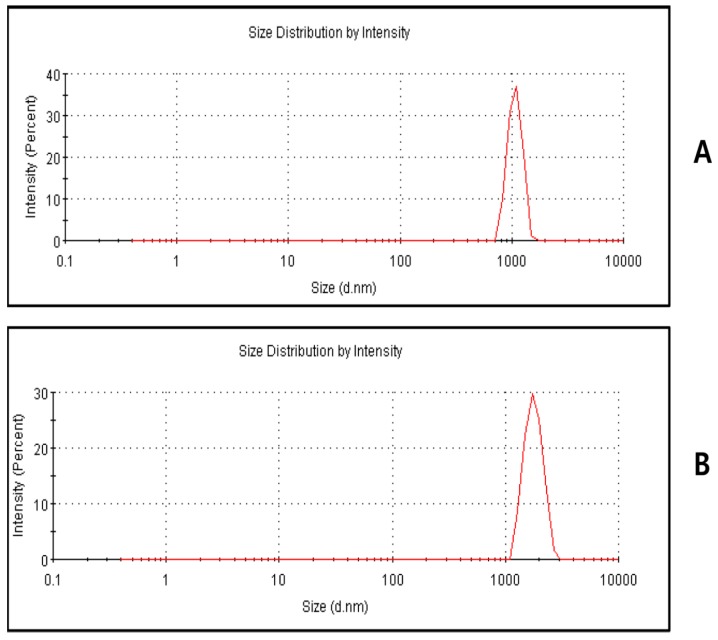
Size distribution analysis of GO and UA-rGO. Aqueous dispersions of GO (**A**) and UA-rGO (**B**) at 250 μg/mL were characterized by DLS analysis at the scattering angle θ =90° using a particle size analyzer. The data show the average values from triplicate measurements.

Characterization of nanoparticles in solution using DLS is an important aspect of *in vitro* toxicity assessment [28,49,56]. As shown in Figure 4A,B, it is clear that the particle sizes of GO and UA-rGO were 1180 ± 50 nm (PDI 0.1) and 1780 ± 70 nm (PDI 0.156), respectively. This suggests that the particle size of UA-rGO was increased after reduction. Liu *et al.* [23] evaluated the particle size of aqueous dispersions of Gt, GtO, GO, and rGO using DLS; the sizes were 5250, 4420, 560, and 2930 nm, respectively. This demonstrated that the particle size of rGO was larger than GO. Gurunathan *et al.* [28] found that the size of *Bacillus marisflavi* biomass reduced B-rGO (3400 nm) is greater than GO (525 nm). In contrast, the particle size of *Pseudomonas aeruginosa*-reduced GO was 2676 nm rGO than GO 453 nm [28]. Stankovich *et al.* [45] reported that functionalized graphene nanoplates treated with isocyanate produce an average size distribution of about 560 ± 60 nm. Humanin, a protein-reduced GO, is slightly larger than previously reported; the average hydrodynamic diameters of GO and humanin reduced GO (HN-rGO), obtained under the same instrumental conditions, were 7800 ± 50 nm and 9800 ± 70 nm, respectively [16]. To confirm further the lateral size, SEM analysis was performed in the dispersions of GO and UA-rGO.

### 2.5. Surface Analysis of GO and UA-rGO

**Figure 5:**
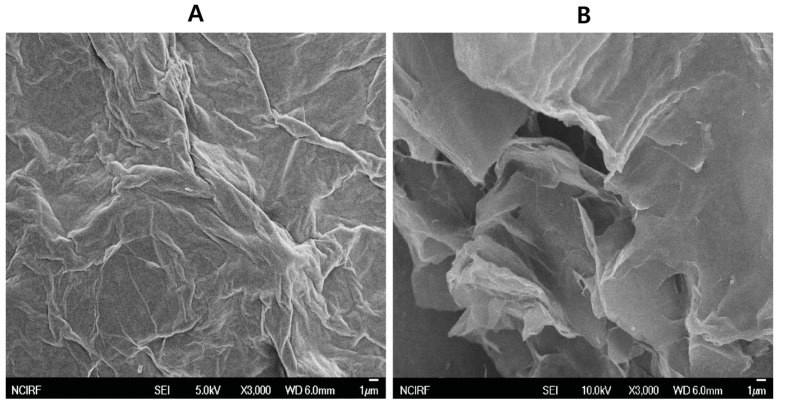
SEM images of GO and UA-rGO. Representative SEM images of GO (**A**) and UA-rGO (**B**) dispersions at 500 μg/mL.

The microstructure and surface analysis of GO and UA-rGO was performed using SEM. Shown in Figure 5A are typical SEM images of GO. GO has a sponge-like morphology with a multi-layer, typically disordered sheet-like structure, with the graphene sheets covered with porous carbon [57]. In addition, it shows a typical crumpled and wrinkled graphene sheet structure on the rough surface of the film, which was the result of deformation upon the exfoliation and restacking processes [58]. The morphology resembles a thin curtain. These parameters indicate very good exfoliation of Gt during the oxidation process. Figure 5B shows a typical SEM image of the UA-rGO, revealing the morphology of the graphene sheets, which exhibit a typical wrinkled structure [9] with the corrugation and scrolling that are fundamental to graphene [59]. The GO sheets had more stacking of sheets, but the UA-rGO were more separated flakes, resembling good lamellar structure, and rich wrinkles on the surface of graphene [60]. Furthermore, UA-rGO exhibits a smooth, homogeneous surface and the typical wrinkled structure that caused sheet folding [61]. Our results are consistent with high quality rGO nanosheets that were prepared from natural Gt through oxidation followed by the solvothermal reduction method [58]. Fu *et al.* [58] observed that the rGO nanosheets are layer structured, irregular and folding, and entangled with each other. They also suggested that the single- or few-layer rGO nanosheets had many wrinkles. Wang *et al.* [62] reported that corrugation and scrolling represent the intrinsic nature of graphene, because the 2D membrane structure would be thermodynamically stable via blending [62].

### 2.6. Raman Spectroscopy Analysis of GO and UA-rGO

**Figure 6:**
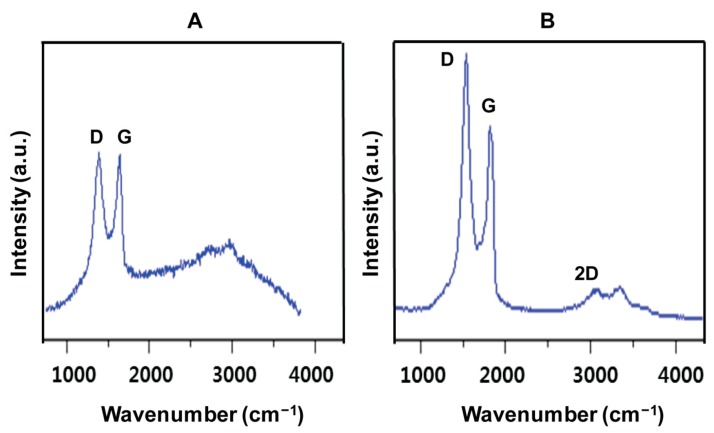
Raman spectroscopy analyses of GO and UA-rGO samples. Raman spectra of GO (**A**) and UA-rGO (**B**) were obtained using laser excitation of 532 nm at the power of 1 mW, after the removal of background fluorescence. The intensity ratios of the D-peak to G-peak were 1.5 and 2.02 for GO and UA-rGO, respectively. At least three independent experiments were performed for each sample and reproducible results were obtained.

Raman spectroscopy is widely used to evaluate the crystal structure, disorder, and defects in graphene-based materials. Raman scattering is very sensitive to the microstructure of nanocrystalline materials [63]. The Raman spectra of GO and UA-rGO are presented in Figure 6A,B, respectively. A spectrum of GO shows that the D and G band positions are located at 1355 and 1608 cm^−1^, whereas UA-rGO located at 1351 and 1599 cm^−1^. The reduction process used for GO is indicated in Raman spectra by the changes in relative intensity of two main peaks: D and G [64]. The D peak was located at 1355 cm^−1^ for GO and at 1351 cm^−1^ for UA-rGO, the changing position indicates that from a defect-induced breathing mode of sp^2^ rings [65], which is common to all sp^2^ carbon lattice and arises from the stretching of C-C bond. The G peak located at around 1608 cm^−1^ for GO and at 1599 cm^−1^ for UA-rGO is due to the first order scattering of the E2g phonon of sp^2^ C atoms [65]. The intensity of the D band is related to the size of the in plane sp^2^ domains [66]. The increase of the D peak intensity indicates formation of more sp^2^ domains. The relative intensity ratio of both peaks (ID/IG) is a measure of disorder degree and is inversely proportional to the average size of the sp^2^ clusters [66,67]. The relative intensity of D/G was increased after reduction by UA, and the D/G intensity ratio for rGO (2.02) is larger than that for GO (1.5), indicating that new graphitic domains are formed, and the sp^2^ cluster number is increased [65]. The possible reason for increase the intensity ratio of D/G could be the reduction of GO causes fragmentation and yields smaller RGO graphitic domains with different sizes or the recovery of graphitic electronic conjugation for rGO. In addition, it also could possible that UA-rGO more defective and disordered sites which act as active sites for the adsorption of foreign molecules. Our results are consistent with chemically-reduced GO [45], *E. coli* bacteria reduced-GO to graphene [53,67], fungi extracts [39], and plant extract-mediated reduction of GO [49].

### 2.7. UA-rGO Inhibits Cell Viability of Ovarian Cancer Cells

**Figure 7:**
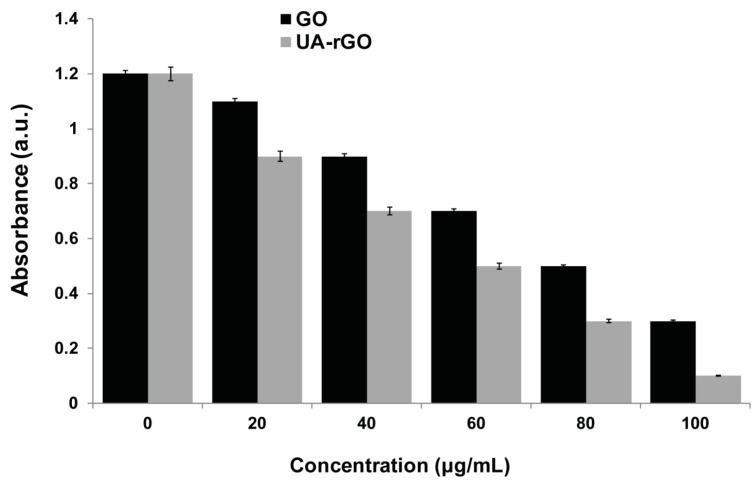
Effects of GO and UA-rGO on the viability of human ovarian cancer cells. The viability of A2780 human ovarian cancer cells was determined after 24 h exposure to different concentrations of GO or UA-rGO using the WST-8 assay. The results are expressed as the mean ± standard deviation of three independent experiments. There was a significant difference in the viability of GO- and UA-rGO-treated cells compared to the untreated cells by the Student’s *t*-test (*p* < 0.05).

Although GO and graphene have been studied extensively, the comprehensive study is limited. To assess the effect of GO and UA-rGO on cell viability, A2780 human ovarian cancer cells were incubated with different concentrations of GO and UA-rGO (20–100 μg/mL) for 24 h. As shown in Figure 7, both GO and UA-rGO decreased the viability of A2780 cells in a dose-dependent manner. However, UA-rGO inhibited the cell viability significantly compared to GO. GO induced cell death in less than 70% of A2780 cells even at the highest tested concentration. In contrast, UA-rGO caused significant cytotoxicity at 20 μg/mL, inducing cell death in 15% of cells, and at 100 μg/mL, more than 90% of the cells were dead. Hu *et al.* [22] found that rGO nanosheets with a thickness of 4.6 μm reduced cell viability to 47% and 15% at concentrations of 20 μg/mL and 85 μg/mL, respectively. The initial finding by Chang *et al.* [26] demonstrated that the lowest concentration of GO that induces toxicity produces neither obvious cellular uptake nor obvious effects on the morphology, viability, mortality, and membrane integrity in adenocarcinoma human alveolar basal epithelial (A549) cells; however, it can induce oxidative stress at a concentration as low as 10 mg/mL [26]. Lammel *et al.* [68] extensively studied the toxicity of GO in HepG2 cell line using four different cell viability assays. They concluded that GO caused a dose-dependent decrease in the cell viability, indicating plasma membrane damage; loss of plasma membrane structural integrity was associated with a strong physical interaction of GO with the phospholipid bilayer. The penetration of GO into the cells through the plasma membrane resulted in altered cell morphology and an augmented number of apoptotic cells [68]. In contrast to GO, studies on the effect of rGO and graphene are limited in human cells. Graphene sheets and reduced GO nanoplatelets (rGONPs) affected the viability of human mesenchymal stem cells in a concentration- and size-dependent manner [69]. Jaworski *et al.* [70] have reported that graphene platelets at a concentration of 100 μg/mL reduced the viability of human glioblastoma U87 and U118 cells to 54% and 60%, respectively. Gurunathan and co-workers found that the toxicity of bacterially-reduced and resveratrol-reduced GO is dose-dependent in human breast cancer cells [28] and ovarian cancer cells [18], respectively.

### 2.8. Effect of UA-rGO on Cell Morphology

**Figure 8:**
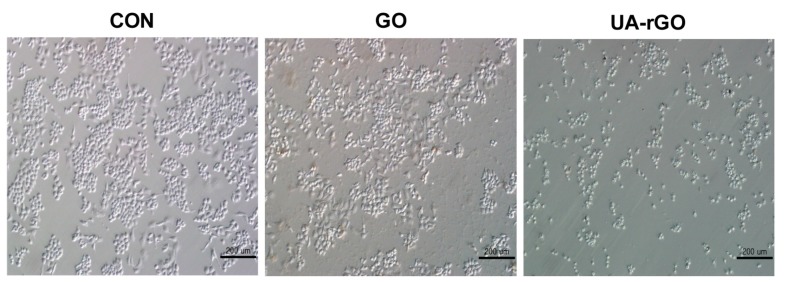
Morphology of human ovarian cancer cells treated with GO and UA-rGO. The morphology of A2780 cells treated with GO and UA-rGO (50 µg/mL) for 24 h. The images were produced by interference contrast light microscopy.

To further explore the cytotoxicity of GO and UA-rGO, the cells were treated with GO or UA-rGO and the effect on the morphology of A2780 cells was examined by light microscopy. The results showed that GO and UA-rGO induced significant morphological changes, including the loss of cell shape, disruption of cell monolayers, and reduction of cell adhesion, indicative of impaired cell viability [18]. Figure 8 shows the morphology of A2780 cell monolayers incubated without (control) or with 50 µg/mL GO or UA-rGO for 24 h. The analysis revealed a significant difference between control and UA-rGO-treated cells. Control A2780 monolayers appeared as oval-shaped, dense, small round clumps with indistinct cellular borders, whereas UA-rGO-treated cells had severe damage in cell structure, characterized by lack of distinct cellular boundaries, elongation, scattering, and reduced cell numbers compared to the untreated cells. These results are in agreement with those reported by Jaworski *et al.* [71] on the effect of rGO treated U87 glioma cells and resveratrol-reduced GO in ovarian cancer cells [18]. The curcumin-reduced GO sheet exhibited significant toxicity and/or cell morphological changes at 70 μg/mL and above in MDA-MB-231 and SKBR3 cell lines [72].

### 2.9. Effect of UA-rGO on Membrane Integrity

**Figure 9:**
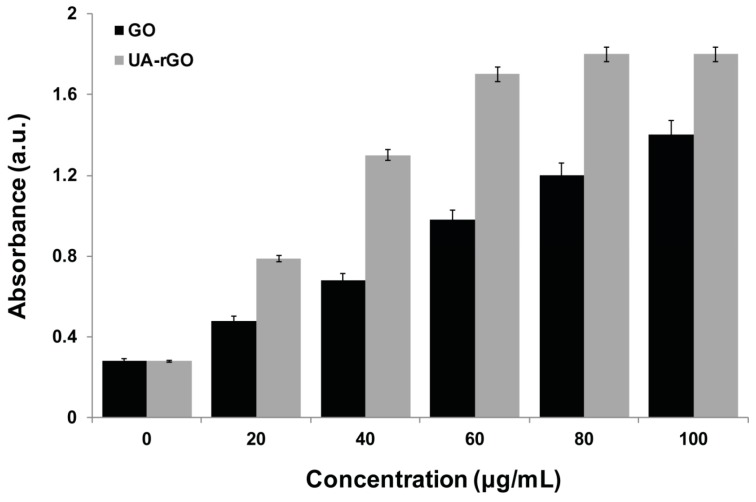
GO and UA-rGO induce release of LDH to the culture supernatant of human ovarian cancer cells. LDH activity was measured at 490 nm using the LDH cytotoxicity kit. The results are expressed as the mean ± standard deviation of three independent experiments. There was a significant difference in LDH activity of GO- and UA-rGO-treated cells compared to the untreated cells by the Student’s *t*-test (*p* < 0.05).

Membrane integrity, which is an important factor determining cell survival, was analyzed by the release of intracellular lactate dehydrogenase (LDH) molecules into the culture medium [18,70]. To determine the LDH activity, the cells were treated with GO and UA-rGO for 24 h. The results showed that GO and UA-rGO caused dose-dependent LDH release into the A2780 cell culture supernatant (Figure 9), suggesting the disruption of the cell membrane.

The treatment with UA-rGO caused a more pronounced negative effect on the membrane integrity of A2780 cells than treatment with GO, which corroborates with the results of the cell viability assay. Zhang *et al.* [73] performed a comparative analysis of mitochondrial toxicity and cell membrane integrity in neuronal PC12 cells with graphene and single-wall CNTs (SWCNT). The treatment with graphene and SWCNT for 24 h decreased the metabolic activity of PC12 cells in a dose-dependent manner. Interestingly, graphene produced higher toxicity at low concentrations than SWCNT. The highest concentration of graphene (100 μg/mL) significantly increased LDH release and the generation of ROS and caspase-3 activation. Yan *et al.* [74] showed minimal toxicity of GO in human retinal pigment epithelium (ARPE-19) cells by analyzing various parameters such as cell morphology, viability, membrane integrity, and apoptosis. Schinwald *et al.* [75] reported that the immortalized human acute monocytic leukemia cells (THP-1) treated with graphene platelets had a strong tendency to localize close to the cells. At a concentration of 100 μg/mL, a 24 h treatment resulted in 50% cell death and loss of membrane integrity and apoptosis. Wang *et al.* [76] have demonstrated that GO caused cytotoxicity and genotoxicity in human lung fibroblast cells, which correlated with its effects on cell membrane integrity and depended on particle size, shape, composition, surface charge, and chemistry. The toxicity of UA-rGO is consistent with bacterially-reduced GO, which exhibited significant toxicity to MCF-7 cells in a dose-dependent manner and increased leakage of LDH [28] and hydrazine-reduced GO in ovarian cancer cells [18].

### 2.10. UA-rGO Induces ROS Generation

**Figure 10:**
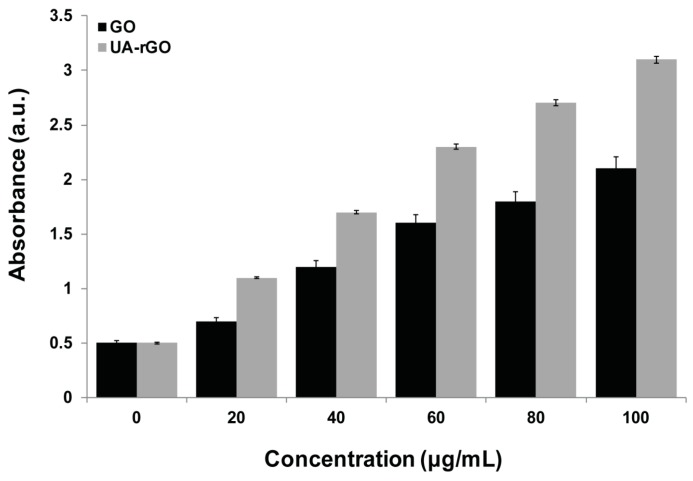
GO and UA-rGO induce ROS generation in human ovarian cancer cells. Relative fluorescence of 2′,7′-dichlorofluorescein was measured at the excitation of 485 nm and emission of 530 nm using a spectrofluorometer. The results are expressed as the mean ± standard deviation of three independent experiments. The treated groups showed statistically significant differences from the control group by the Student’s *t*-test (*p* < 0.05).

A recent study described that ROS plays a significant role in apoptosis, gene expression, and the activation of cell signaling cascades; in addition, it serves as both intra- and intercellular messengers [77]. Mitochondria are an important source of ROS, and electrons derived from the oxidation of metabolic intermediates can lead to the generation of ROS at specific sites in mitochondria within most mammalian cells [42,78]. Therefore, to determine the effect of UA-rGO on ROS generation, the ovarian cells were treated with GO and UA-rGO for 24 h. We then measured the level of ROS using intracellular peroxide-dependent oxidation of 2′,7′-dichlorodihydrofluorescein diacetate (DCFH-DA). As shown in Figure 10, GO or UA-rGO induced ROS generation in a concentration-dependent manner, significantly higher than control cells. The effect of UA-rGO was stronger than that of GO, similar to other cytotoxicity parameters [18,79]. Studies from Li *et al.* [80] and Sasidharan *et al.* [81] demonstrated that pristine graphene could increase ROS and apoptosis in murine RAW 264.7 macrophages via the depletion of mitochondrial membrane potential (MMP). The toxicity of UA-rGO in ovarian cancer cells could be due to the possible generation of ROS, similar to that of other nanomaterials such as multi-wall carbon nanotubes [82], and silver nanoparticles [79] in other human cells. The increased ROS level induced by GO and UA-rGO is in agreement with the results of the WST-8 and LDH assays, suggesting that GO and UA-rGO induce cytotoxicity via oxidative stress [18]. Evidence from Lammel *et al.* [68] suggests that GO and carboxyl graphene nanoplatelets induce ROS in human hepatocellular carcinoma HepG2 cells in a concentration- and time-dependent manner. Akhavan *et al.* [30] demonstrated the size- and concentration-dependent cytotoxicity and genotoxicity of rGO and GO nanoplatelets in human mesenchymal stem cells (hMSCs) and RNA efflux from cells, which is an indirect indicator of membrane damage. Interestingly, they also found that the smaller-sized-rGO induced higher RNA effluxes than did the larger-sized-rGO sheets. Moreover, rGOs generated 13–26 fold higher levels of ROS than the control [30]. Taken together, this suggests that oxidative stress is one of the key mechanisms involved in rGO cytotoxicity.

### 2.11. UA-rGO Activates Caspase-3 Activity

**Figure 11:**
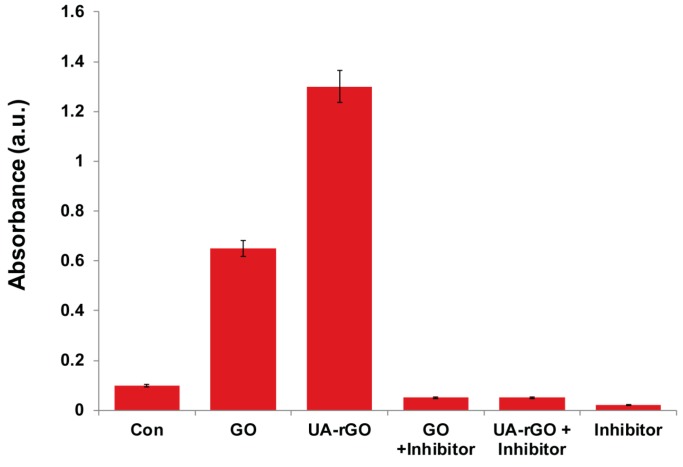
GO and UA-rGO induce caspase-3 activity in human ovarian cancer cells. Ovarian cancer cells were treated with GO or UA-rGO with or without caspase-3 inhibitor Ac-DEVD-CHO for 24 h. The concentration of p-nitroanilide released from the substrate was calculated from the absorbance at 405 nm. The results are expressed as the mean ± standard deviation of three separate experiments. The treated groups showed statistically significant differences from the control group by the Student’s *t*-test (*p* < 0.05).

ROS plays an important role in the activation of cell death through the formation of the mitochondrial permeability transition pore (MPTP), which can activate mitochondria-initiated cell death pathways, including caspase-dependent and caspase-independent pathways [18,83,84]. The caspase family is involved in important biological processes such as inflammation, cell differentiation, proliferation, cell cycle regulation, cell division, and fusion [85]. An execution pathway is initiated by the cleavage of caspase-3, resulting in DNA fragmentation, degradation of cytoskeletal and nuclear proteins, crosslinking of proteins, formation of apoptotic bodies, and expression of ligands for phagocytic cells [86]. Among several caspases, caspase-3 is the executioner, it has a significant role in mediating apoptosis among the caspase family members [86,87,88]. To evaluate a role for caspase activity, we investigated the UA-rGO effect on activation of caspase-3. The cells were treated with 50 μg/mL of GO or UA-rGO for 24 h. For each group, the cells were treated with or without a caspase inhibitor DEVD-CHO. After 24 h treatment, caspase-3 activity was measured using a spectrophotometric assay. Figure 11 illustrates the increase in the levels of caspase-3 activity induced by GO or UA-rGO treatment. In the presence of the caspase-3 inhibitor, caspase-3 activity matched that of the untreated cells, suggesting that graphene may induce apoptosis in ovarian cancer cells in a caspase-3-dependent manner. GO and UA-rGO both stimulated a significant increase in caspase-3 activity (6-fold and 13-fold, respectively), but treatment with UA-rGO showed a significant increase compared to GO. Graphene stimulates both ROS generation and caspase-3 activation in a concentration-and time-dependent manner, indicating the induction of apoptosis [73]. Similarly, Li *et al.* [80] demonstrated that pristine graphene is able to activate apoptosis in macrophages through the involvement of caspase-3. Our results and previously reported data suggest that graphene nanoparticles may induce apoptotic cell death via activation of caspase-3.

### 2.12. UA-rGO Activates DNA Fragmentation

**Figure 12:**
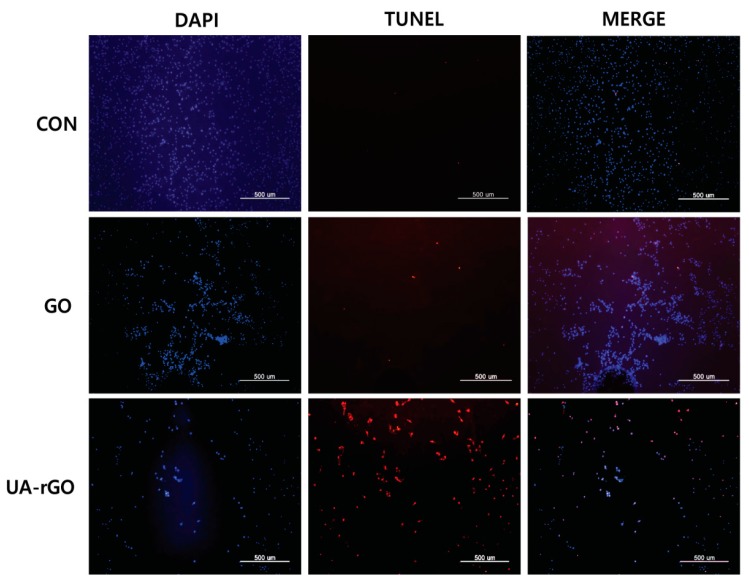
GO and UA-rGO induce apoptosis in human ovarian cancer cells. Apoptosis of human ovarian cancer cells after 24 h treatment with 20 µg/mL of GO and UA-rGO was assessed by the TUNEL assay; the nuclei were counterstained with DAPI. Representative images show apoptotic (fragmented) DNA (red) and corresponding nuclei (blue).

Recently, caspase-3 has been specifically implicated as the effector caspase responsible for cleavage of the human DNA fragmentation factor (DFF) and the inhibitor of the murine caspase-activated DNase [89,90,91]. Caspase-3 has also been shown to be necessary for the typical morphologies associated with apoptosis. To determine the effect of UA-rGO on the extent of apoptosis in ovarian cancer cells, we assessed the execution of apoptotic events that precede chromosomal DNA fragmentation, which is a hallmark of apoptosis [18]. The cells were treated with GO and UA-rGO, and assayed by TUNEL. Treatment with UA-rGO resulted in a significant number of TUNEL-positive cells, whereas in the control, no apoptotic cells were observed (Figure 12). In contrast to UA-rGO-treated cells, GO did not induce significant DNA fragmentation. Carbon nanomaterials such as nanodiamonds and multi-walled carbon nanotubes can induce the expression of chromosomal DNA-damage biomarkers p53, MOGG-1, and Rad51 through generation of ROS [92,93,94]. Akhavan *et al.* [30] performed a genotoxicity study demonstrating that rGO, having average lateral dimensions of 11 nm and 91 nm, initiated significant increases in DNA damage and chromosomal aberration frequency at concentrations as low as 0.1 mg/mL and 1.0 mg/mL, respectively. The results from Akhavan and co-workers suggest that the interaction of rGOs with hMSCs, and probably other cells, strongly depends on their lateral size. The possible mechanism of rGO-induced toxicity in DNA fragmentation occurs via oxidative stress and direct contact of the sharp edges with the cells [30]. Jaworski and co-workers [70] demonstrated the toxicity of various carbon materials such as GN, rGO, Gt, ultra-dispersed detonation diamond (UDD), and GO by assessing cell viability and DNA fragmentation. They concluded that, with the exception of GO, all other carbon materials show genotoxic effects in glioblastoma cancer cells U87 [70,95]. Daunorubicin-loaded graphene-gold nanocomposites induce apoptosis by activating caspases 8 and 3 and inhibit the growth of multidrug-resistant leukemia cells *in vivo* [96]. Taken together, the data suggest that graphene nanoparticles can induce DNA fragmentation in cancer cells.

## 3. Materials and Methods

### 3.1. Materials

Gt powder, NaOH, KMnO_4_, NaNO_3_, anhydrous ethanol, 98% H_2_SO_4_, 36% HCl, 30% H_2_O_2_, resveratrol, fetal bovine serum (FBS), UA, and the *in vitro* toxicology assay kit were purchased from Sigma-Aldrich (St Louis, MO, USA). Penicillin-streptomycin, trypsin-EDTA, Dulbecco’s Modified Eagle Medium (DMEM), and 1% antibiotic-antimycotic solution were obtained from GIBCO (Life Technologies, Carlsbad, CA, USA). All other chemicals were purchased from Sigma (St. Louis, MO, USA) unless stated otherwise.

### 3.2. GO Synthesis

GO was synthesized according to the method described earlier [97] with some modifications [18,44]. In a typical synthesis process, 2 g of natural Gt powder was added to 350 mL of cooled (0 °C) H_2_SO_4_, and then 8 g of KMnO_4_ and 1 g of NaNO_3_ were added gradually while stirring. The mixture was transferred to a 40 °C water bath and stirred for 60 min. Deionized water (250 mL) was slowly added, and the temperature was increased to 98 °C. The mixture was maintained at 98°C for 30 min, and the reaction was terminated by the addition of 500 mL deionized water and 40 mL 30% H_2_O_2_. The color of the mixture changed to brilliant yellow, indicating the oxidation of pristine Gt to GO. The mixture was then filtered and washed with diluted HCl to remove metal ions. Finally, the product was washed repeatedly with distilled water until pH 7.0 was achieved, and the synthesized GO was sonicated for 60 min.

### 3.3. Preparation of UA-rGO

GO reduction was performed as described previously [18,44,49] with some modifications. UA-rGO was obtained using resveratrol both as a reducing agent and as a stabilizer. In a typical procedure, 1 mg UA was mixed with 1 mg/mL GO, sonicated for 15 min, and incubated at 40 °C for 1 h. The mixture was then cooled to room temperature and sonicated for an additional 15 min. After vigorous stirring for 5 min, the mixture was stirred in a 90 °C-water bath for 1 h. The resulting stable black dispersion was then centrifuged and washed with water three times, producing a homogenous UA-rGO suspension without aggregation. The resulting UA-rGO sheets were redispersed in water before further use.

### 3.4. Characterization of GO and UA-rGO

GO and UA-rGO were characterized as described previously [18,24,44,49]. UV-visible spectra were recorded using OPTIZEN POP spectrophotometer (Mecasys Co., Seoul, Korea). XRD analysis was performed in a Bruker D8 DISCOVER X-ray diffractometer (Bruker AXS GmBH, Karlsruhe, Germany). The X-ray source was 3 kW with a Cu target, and high-resolution XRD patterns were measured using a scintillation counter (*λ* = 1.5406 Å). XRD was run at 40 kV and 40 mA, and samples diffraction was recorded at 2θ values between 5° and 50°. Dry GO or UA-rGO powder was suspended in KBr, and Fourier transform infrared spectroscopy (FTIR) was performed using a Spectrum GX spectrometer (Perkin Elmer Inc., Waltham, MA, USA) within the range of 500–4000 cm^−1^. Particle sizes of the GO and UA-rGO dispersions were measured using a ZetasizerNano ZS90 instrument (Malvern Instruments, Worcestershire, UK). A JSM-6700F semi-in-lens field emission scanning electron microscope (JEOL, Tokyo, Japan) was used to acquire SEM images. Solid samples were transferred to a carbon tape in an SEM sample holder, and the analysis was performed at an average working distance of 6 mm. GO and UA-rGO Raman spectra were measured using a WITEC Alpha300 laser (Ulm, Germany) at the wavelength of 532 nm. Calibration was initially performed using an internal silicon reference at 500 cm^−1^, which gave a peak resolution of less than 1 cm^−1^. The spectra were measured from 500 to 4500 cm^−1^. All samples were deposited onto glass slides in powdered form without using solvent.

### 3.5. Cell Culture and Exposure to GO and UA-rGO

Cell culture was performed as described previously [18,28]. Human ovarian A2780 cancer cells were cultured in DMEM, supplemented with 10% FBS, 2mM glutamine, and 100 U/mL penicillin-streptomycin, in a humidified 5% CO_2_-incubator at 37 °C. The medium was replaced three times a week, and cells were passaged at sub-confluency. At approximately 75% confluence, cells were harvested using 0.25% trypsin-EDTA and seeded in 75-cm^2^ flasks, 6-well plates, or 96-well plates depending on the experiment. After 24 h, the medium was replaced with fresh medium containing GO or UA-rGO at different concentrations (20–100 µg/mL); cells not exposed to GO or UA-rGO served as the negative control. After 24 h incubation, the treated cells were analyzed for viability, LDH release, and ROS generation.

### 3.6. Cell Viability Assay

Cell viability was examined using the WST-8 assay as described previously [28,98]. Typically, 1 × 10^4^ cells were seeded in 100 μL of 10% FBS-containing DMEM in a 96-well plate. After 24 h, the cells were washed with 100 μL of serum-free DMEM two times and incubated in 100 μL of serum-free DMEM containing different concentrations of GO or UA-rGO suspensions. After 24 h of exposure, the cells were washed twice with serum-free DMEM, and 15 μL of the WST-8 solution was added to each well containing 100 μL of serum-free DMEM. After a 1 h incubation, 80 μL of the mixture was transferred to another 96-well plate because residual GO or UA-rGO can affect the absorbance measured at 450 nm using a micro plate reader. Cell-free control experiments were performed to determine whether GO and UA-rGO directly reacted with the WST-8 reagent. For this, resveratrol (0–200 µM) or 100 μL of GO or UA-rGO suspensions (20–100 μg/mL) were incubated with 10 μL of the WST-8 reagent in a 96-well plate for 1 h. The plates were centrifuged to precipitate GO and UA-rGO, and 100 μL of the supernatant was transferred to another 96-well plate to measure optical density at 450 nm.

### 3.7. Cell Morphology

Ovarian cancer cells were plated in 6-well plates (1 × 10^4^ cells per well) in 10% FBS-containing DMEM. After 24 h, medium was changed to serum-free DMEM with or without 20 μg/mL GO or UA-rGO and incubated for 24 h. Cell morphology was examined using an OLYMPUS IX71 microscope (Olympus Corporation, Tokyo, Japan) with appropriate filter sets.

### 3.8. Membrane Integrity

The cell membrane integrity of human ovarian cancer cells (A2780) was evaluated by determining LDH activity in cell supernatants according to the manufacturer’s instructions (*In Vitro* Toxicology Assay kit; Sigma) and as described previously [18]. Briefly, cells were exposed to various concentrations of GO or UA-rGO (20–100 μg/mL) in triplicate for 24 h; 100 μL of each cell-free supernatant was transferred to a new 96-well plate and mixed with 100 μL of the LDH reagent. After a 3 h incubation under standard conditions, optical density was determined at 490 nm using a micro plate reader.

### 3.9. Determination of ROS

Intracellular ROS was measured based on the intracellular peroxide-dependent oxidation of 2′,7′-dichlorodihydrofluorescein diacetate (DCFH-DA, Molecular Probes, USA) to a fluorescent compound 2′,7′-dichlorofluorescein (DCF), as previously described [28,29]. Cells were seeded onto 24-well plates at a density of 5 × 10^4^ cells per well and cultured for 24 h. After washing twice with PBS, fresh medium containing different concentrations of GO or UA-rGO (20–100 μg/mL) was added, and the cells were incubated for 24 h. The cells were then supplemented with 20 μM DCFH-DA and incubation continued for 30 min at 37 °C. Cells were pretreated with *N*-acetylcysteine (NAC) to a final concentration of 1 mM. The positive control for ROS generation was obtained by the addition of H_2_O_2_ to a final concentration of 3 mM and AgNPs (10 μg/mL). The cells were rinsed with PBS, 2 mL of PBS was added to each well, and fluorescence intensity was determined using a Gemini EM spectrofluorometer (Molecular Devices, Sunnyvale, CA, USA) with the excitation at 485 nm and emission at 530 nm.

### 3.10. Measurement of Caspase-3 Activity

Caspase-3 activity was assayed as described earlier [99] using a commercial kit (Sigma) according to the manufacturer’s instructions. Cancer cells were plated as above and treated with GO or UA-rGO (50 μg/mL) or not treated (control), and each group was treated with or without caspase-3 inhibitor for 24 h. The cells were washed with ice-cold PBS and lysed with 100 μL of lysis buffer (50 mM Tris-HCl, pH 7.5, 150 mM NaCl, 1 mM EGTA, 1 mM NaF, 1% Nonidet P-40, 1mM PMSF, and protease inhibitor cocktail) for 30 min at 4 °C. The extracts were collected after centrifugation at 10,000 rpm for 10 min, and the protein concentration was determined using the Bio-Rad protein assay kit (Bio-Rad, Hercules, CA, USA). Equal amounts (50 μg) of protein extracts were mixed with assay buffer (20 mM HEPES, pH 7.4, 100 mM NaCl, 0.1% CHAPS, 10 mM DTT, 1 mM EDTA, 10% sucrose), added to 96-well plates, and incubated with caspase-3 substrate acetyl-Asp-Glu-Val-Asp p-nitroanilide (Ac-DEVD-pNA) and caspase-3 inhibitor (Ac-DEVD-CHO) for 1 h and the absorbance was measured at 405 nm. The colorimetric assay is based on the hydrolysis of caspase-3 substrate by caspase-3, resulting in the release of the p-nitroaniline (pNA).

### 3.11. TUNEL Assay

Apoptosis induced by UA-rGO was measured by TUNEL assay using the DNA fragmentation Imaging Kit (Hoffman-La Roche Ltd., Basel, Switzerland) as per manufacturer’s instruction. Briefly, A2780 cells were seeded in 6-well plates (1.5 × 10^6^ cells/well) for 24 h, and then treated with 20 μg/mL of GO or UA-rGO for another 24 h. Cells were detached with trypsin-EDTA, placed on 0.01% polylysine-coated slides, fixed with 4% methanol-free formaldehyde solution, and stained using terminal deoxynucleotidyltransferase and fluorescein-labeled dUTP for fluorescence-based detection of cells containing DNA breaks; cells were counterstained with DAPI to evaluate total cell number. Stained cells were observed using an Axiovert epifluorescence microscope (Axiovert, Carl Zeiss Meditec AG, Jena, Germany) equipped with a triple band-pass filter. To determine the percentage of apoptotic cells, 1000 cells were counted in each experiment. Merged images of TUNEL- and DAPI-stained cells were observed using a fluorescence microscope (OLYMPUS, Tokyo, Japan) at ×500 magnification.

### 3.12. Statistical Analyses

The results are presented as the mean ± SD of at least three independent experiments; all assays were performed in triplicate. The data was analyzed using the Student's *t*-test, and the difference was considered statistically significant at *p*-value less than 0.05.

## 4. Conclusions

Graphene, a one-atom-thick planar sheet of carbon atoms densely packed in a honeycomb crystal lattice, shows unique physical, chemical, and biocompatible properties. Therefore, synthesis of graphene using biological materials has attracted tremendous interest in biomedicine both in academia as well as industry. In this study, we explored the potential for UA, natural human waste, to be utilized for green reduction and functionalization of chemically exfoliated GO. The reduction of GO was confirmed by various analytical techniques such as ultraviolet-visible spectroscopy, XRD, DLS, FTIR, SEM, and Raman spectroscopy. These results suggest that a powerful natural human waste, UA, a reducing and stabilizing agent, can be used as a suitable substitute for hydrazine in large-scale production of graphene. The cytotoxicity of prepared UA-rGO was examined in human ovarian cancer cells with a series of assays. The results show that UA-rGO at the minimum concentration of 20 μg/mL exhibited toxicity; increasing dose of UA-rGO caused higher toxicity. In contrast, GO is less toxic than UA-rGO. In addition, we observed more severe morphological changes in UA-rGO treated cells than in GO-treated cells. Interestingly, compared to GO, UA-rGO showed significant dose-dependent cytotoxicity in A2780 ovarian cancer cells by reducing cell viability, increasing release of LDH, generating ROS, activating caspase-3, and DNA fragmentation. Conventional chemotherapy approaches to cancer are limited by lack of specificity, systemic toxicity, and chemoresistance. At this juncture, nanomaterials such as graphene could provide more specific cancer treatment, effectively reducing undesired side effects and providing accurate diagnosis and effective therapy. However, further detailed and mechanistic studies using animal models are required for theranostic applications.
